# Antifibrotic therapy for idiopathic pulmonary fibrosis: time to treat

**DOI:** 10.1186/s12931-019-1161-4

**Published:** 2019-09-06

**Authors:** Toby M. Maher, Mary E. Strek

**Affiliations:** 1 National Institute for Health Research Respiratory Clinical Research Facility, Royal Brompton and Harefield NHS Foundation Trust, Sydney Street, London, SW3 6NP UK; 2 Fibrosis Research Group, National Heart and Lung Institute, Imperial College, Cale Street, London, SW3 6LY UK; 3 Section of Pulmonary & Critical Care Medicine, The University of Chicago, Chicago, IL USA

**Keywords:** Nintedanib, Pirfenidone, Interstitial lung disease, Therapeutics, Treatment, Mortality

## Abstract

Idiopathic pulmonary fibrosis (IPF) is a progressive disease with a dismal prognosis. The average life expectancy of untreated patients with IPF is only 3 to 4 years. Decline in forced vital capacity (FVC) in patients with IPF appears to be almost linear, with patients with well-preserved FVC at baseline experiencing the same rate of decline in FVC as patients with more advanced disease. Two antifibrotic therapies have been approved for the treatment of IPF: nintedanib and pirfenidone. These drugs slow decline in lung function and reduce the risk of acute respiratory deteriorations, which are associated with very high morbidity and mortality. Individual clinical trials have not been powered to show reductions in mortality, but analyses of pooled data from clinical trials, as well as observational studies, suggest that antifibrotic therapies improve life expectancy. Despite this, many individuals with IPF remain untreated. In many cases, this is because the physician perceives that the disease is stable and so does not warrant therapy, or has concerns over the potential side-effects of antifibrotic drugs. There remains a need to educate pulmonologists that IPF is a progressive, irreversible and fatal disease and that prompt treatment is critical to preserving patients’ lung function and improving outcomes. Most individuals can tolerate antifibrotic therapy, and dose adjustment has been shown to be effective at reducing side effects without compromising efficacy. In addition to anti-fibrotic therapies, individuals with IPF benefit from a holistic approach to their care that includes symptom management and supportive care tailored to the needs of the individual. An animation illustrating the themes covered in this article will be available at: http://www.usscicomms.com/respiratory/maher/treatment-of-IPF.

## Background

**Fig. 1:**
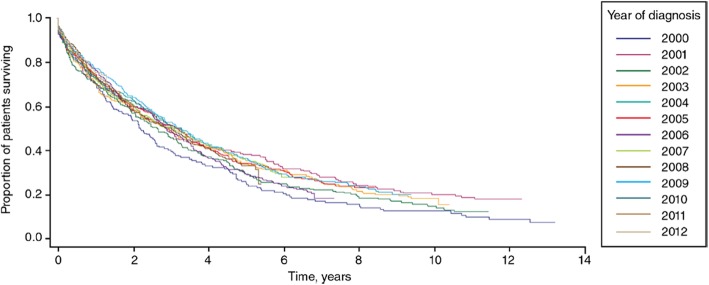
Kaplan-Meier analysis of survival in patients with incident IPF [3]. Population-based study of all incident cases of IPF in the United Kingdom identified using a broad case definition based on diagnostic codes. Republished with permission of Adv Ther, from Incidence, prevalence, and survival of patients with idiopathic pulmonary fibrosis in the UK, Strongman et al., 35, 2018; permission conveyed through Copyright Clearance Center, Inc.

IPF is a progressive and ultimately fatal interstitial lung disease (ILD) characterized by radiologic and/or histopathologic findings of usual interstitial pneumonia [1]. IPF has a poor prognosis, with an average life expectancy in patients not receiving antifibrotic therapy of only 3 to 4 years [2, 3] (Fig. 1). As the disease progresses, lung function declines, accompanied by worsening of dyspnea and functional capacity and deterioration in quality of life [4–6]. Acute exacerbations of IPF (respiratory deteriorations with evidence of new bilateral ground-glass opacification or consolidation on CT) can occur at any time in the course of the disease and are associated with very high mortality [7]. The majority of patients with IPF die from an acute exacerbation or respiratory failure [8, 9]. This article will describe the progressive nature of IPF and the utility of antifibrotic therapies, with a focus on the importance of early treatment.

### Clinical course of IPF

**Fig. 2:**
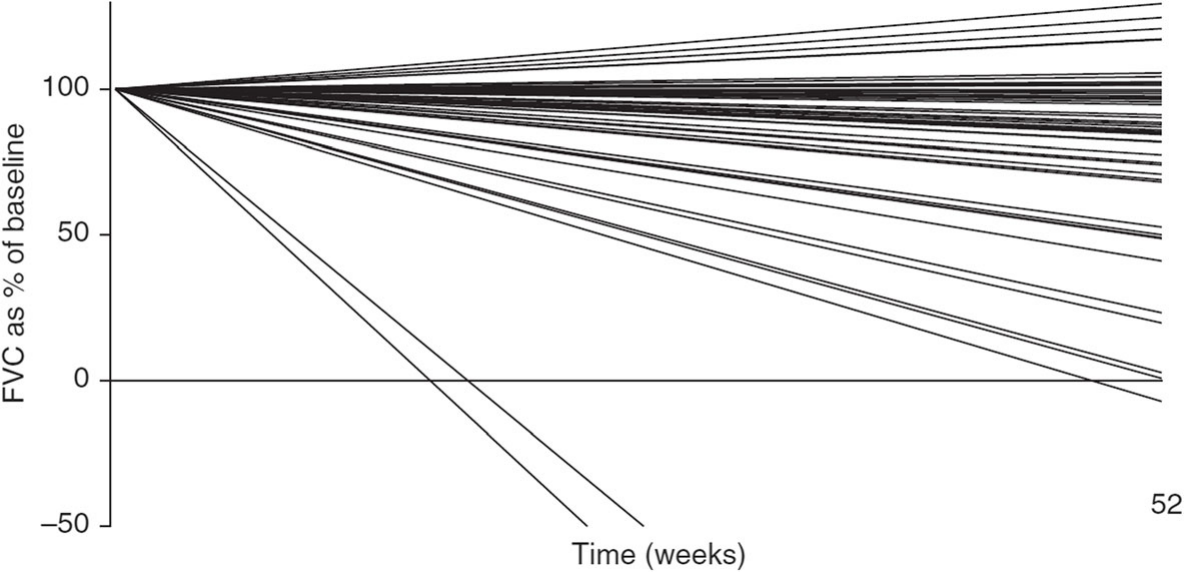
Decline in FVC in patients with IPF based on daily home spirometry [10]. Linear regression lines for every subject were based on all readings obtained between baseline and day 365 without imputation. FVC, forced vital capacity. Reprinted with permission of the American Thoracic Society. Copyright© 2018 American Thoracic Society. Russell AM, et al. 2016. Daily home spirometry: an effective tool for detecting progression in idiopathic pulmonary fibrosis. Am J Respir Crit Care Med 194:989–97. The American Journal of Respiratory and Critical Care Medicine is an official journal of the American Thoracic Society

**Fig. 3:**
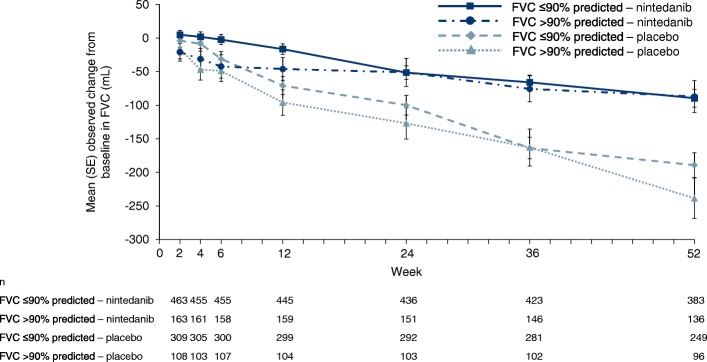
Decline in FVC in subjects with baseline FVC > 90% and ≤ 90% predicted in the INPULSIS trials of nintedanib [13]. Error bars show the standard error. FVC, forced vital capacity. Republished with permission of Thorax, from Nintedanib in patients with idiopathic pulmonary fibrosis and preserved lung volume, Kolb et al., 72, 2017; permission conveyed through Copyright Clearance Center, Inc.

While rates of disease progression are variable between individuals, ultimately lung function declines in all those with IPF. Data from a single-center study based on daily hand-held spirometry suggested that most individuals with IPF show a decline in forced vital capacity (FVC), with only 8% of patients showing stability in FVC over 1 year (Fig. 2) [10]. The results of clinical trials conducted in individuals with IPF and mild or moderate impairment in lung function at baseline suggest an average loss of FVC of 150–200 mL per year in placebo-treated patients [11]. It is important to remember that relative preservation of FVC at baseline does not indicate that FVC will remain stable in the near future [12–14]. Data from the placebo group of the INPULSIS trials showed that subjects with FVC > 90% predicted at baseline experienced the same decline in FVC over the following year as subjects with less well preserved FVC (− 224.6 vs − 223.6 mL/year) (Fig. 3) and that 2.8% of those with FVC > 90% predicted at baseline experienced an acute exacerbation within the next year [13].

### Benefits of antifibrotic therapies

In the US, Europe and many other countries, two drugs are approved for the treatment of IPF: nintedanib and pirfenidone. In vitro studies have shown that by inhibiting signaling mediated via tyrosine kinases, nintedanib inhibits fundamental processes of fibrosis, such as the recruitment, proliferation and differentiation of fibroblasts and fibrocytes and the deposition of extracellular matrix [15]. Data from animal models of fibrosis suggest that nintedanib may also act to normalize the distorted microvascular architecture in the lungs [16]. The mechanism of action of pirfenidone is less well defined, as its target remains unknown, but non-clinical studies suggest that it inhibits pro-fibrotic behaviors in fibroblasts and fibrocytes [17, 18].

**Fig. 4:**
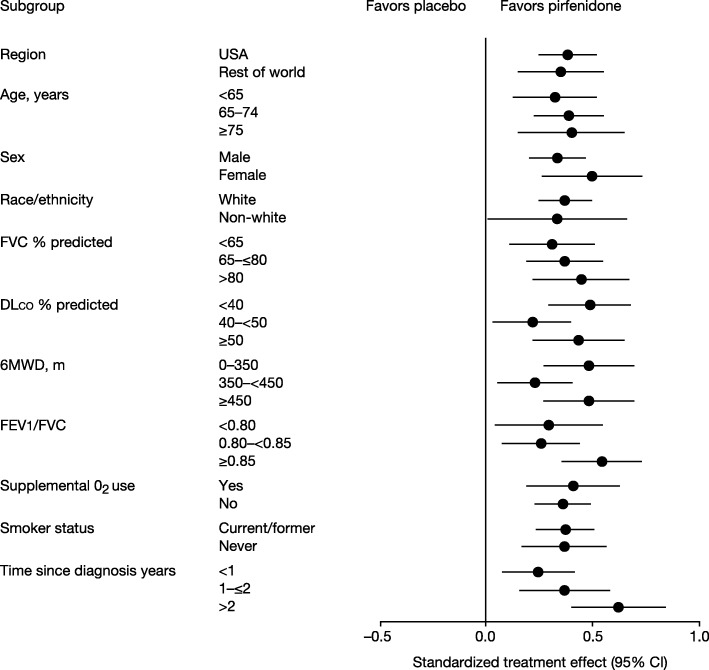
Standardized treatment effect of pirfenidone versus placebo on change in FVC % predicted from baseline to 1 year based on pooled data from the CAPACITY and ASCEND trials (*N* = 1247) [24]. 6MWD, 6-min walk distance; DLco, diffusing capacity of the lungs for carbon monoxide; FEV_1_, forced expiratory volume in 1 s; FVC, forced vital capacity. Error bars show the 95% confidence interval. Reproduced with permission of the © ERS 2018. European Respiratory Journal Jan 2016, 47 (1) 243–253; DOI: 10.1183/13993003.00026-2015. This material has not been reviewed prior to release; therefore the European Respiratory Society may not be responsible for any errors, omissions or inaccuracies, or for any consequences arising there from, in the content

**Fig. 5:**
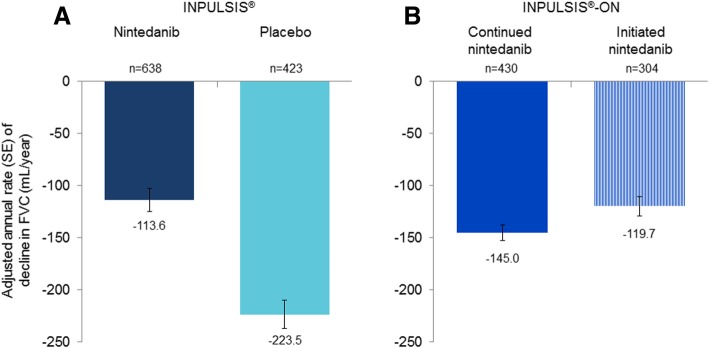
Annual rate of decline in FVC in the INPULSIS trials and their open-label extension INPULSIS-ON [26]. Annual rate of decline in FVC over 52 weeks in INPULSIS and over 192 weeks in INPULSIS-ON. Patients who took nintedanib in an INPULSIS trial continued nintedanib in INPULSIS-ON. Patients who took placebo in an INPULSIS trial initiated nintedanib in INPULSIS-ON. Error bars show the standard error. FVC, forced vital capacity. Republished with permission of Lancet Respir Med, from Long-term treatment with nintedanib in patients with idiopathic pulmonary fibrosis: results from INPULSIS-ON, Crestani et al., doi: 10.1016/S2213-2600(18)30339-4, 2018; permission conveyed through Copyright Clearance Center, Inc.

Clinical trials have demonstrated that nintedanib and pirfenidone reduce the decline in lung function in patients with IPF [19–21], with consistent effects across the spectrum of baseline FVC studied (FVC > 50% predicted) and across subgroups by age, race, gender, and concomitant medication use [13, 22–25] (Fig. 4). The Phase III INPULSIS and ASCEND trials showed that in subjects with mild or moderate FVC impairment at baseline, nintedanib and pirfenidone reduced the rate of decline in FVC by approximately 50% over 1 year of treatment [20, 21]. Further, data from the open-label extension of the INPULSIS trials, INPULSIS-ON, suggest that nintedanib has a sustained effect in reducing decline in lung function over more than 4 years of therapy (Fig. 5) [26]. Recent evidence from the INSTAGE trial suggests that nintedanib has a similar effect on FVC decline in subjects with severe impairment in gas exchange (DL_CO_ ≤ 35% predicted) at baseline as in those with less advanced disease [27]. Although individual clinical trials have not been powered to show significant effects on acute exacerbations and mortality, there is a growing body of evidence that nintedanib and pirfenidone reduce the risk of acute deteriorations in lung function [28, 29] and improve life expectancy [4, 24, 30–37] by reducing the rate at which IPF progresses.

**Fig. 6:**
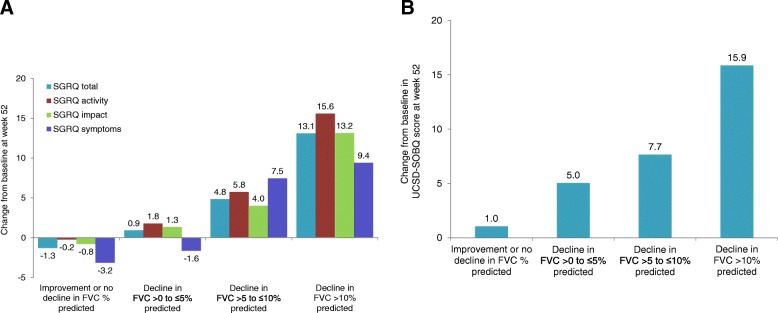
Changes from baseline in SGRQ total and domain scores (A) and UCSD-SOBQ score (B) at week 52 in subgroups by changes in FVC % predicted at week 52 in the INPULSIS trials [40]. SGRQ, St George’s Respiratory Questionnaire; UCSD-SOBQ, University of California San Diego Shortness of Breath Questionnaire; FVC, forced vital capacity

Neither nintedanib nor pirfenidone has been shown in large clinical trials to provide significant relief of the dyspnea, cough, or quality of life impairment associated with IPF. It is unclear as to whether this is because antifibrotic therapies do not have a meaningful effect on symptoms, or because these trials were conducted in patients with mild/moderate impairment in lung function at baseline and lasted only a year. Observational data from clinical practice suggest that antifibrotic therapy may provide a degree of symptom relief [38]. Data from clinical trials [39, 40] and patient registries [5, 41] suggest that greater worsening of FVC is associated with greater worsening in health-related quality of life assessed using patient-reported outcomes such as the St George’s Respiratory Questionnaire (SGRQ), University of California San Diego Shortness of Breath Questionnaire (UCSD-SOBQ) and the cough domains of the Cough and Sputum Assessment Questionnaire (CASA-Q) (Fig. 6). Worsening of FVC is also associated with a reduction in patients’ capacity for exercise [42, 43]. This suggests that over a period of time, reducing the rate at which FVC declines using antifibrotic therapies may reduce the rate at which individuals’ symptoms, functional capacity and quality of life worsen. Further, the relationship between FVC decline and HRQL in people with IPF is unlikely to be linear, such that the same absolute reduction in FVC decline has a greater effect on quality of life later in the course of disease when patients have less physiological reserve remaining following significant loss of lung volume and capacity for gas exchange.

### Importance of prompt treatment of IPF

Diagnosis of IPF is often delayed due to misdiagnosis, with symptoms frequently being ascribed to more common conditions such as COPD, asthma, or cardiac disease, resulting in late referral to specialist centers [6, 36, 44, 45]. This means that by the time a patient receives a diagnosis of IPF, their lung function will have been in decline for some time. It is worth noting that because of the way that predicted values for FVC are calculated (based on a patient’s age, height, and weight), some patients will have an FVC of more than 100% predicted before their lung function begins to decline. Thus it cannot be assumed that a patient with, for example, an FVC of 90% predicted at diagnosis has not already suffered significant loss of lung volume. The presence of comorbid emphysema may also result in FVC being artificially higher than it would have been if the patient had IPF alone [46].

**Fig. 7:**
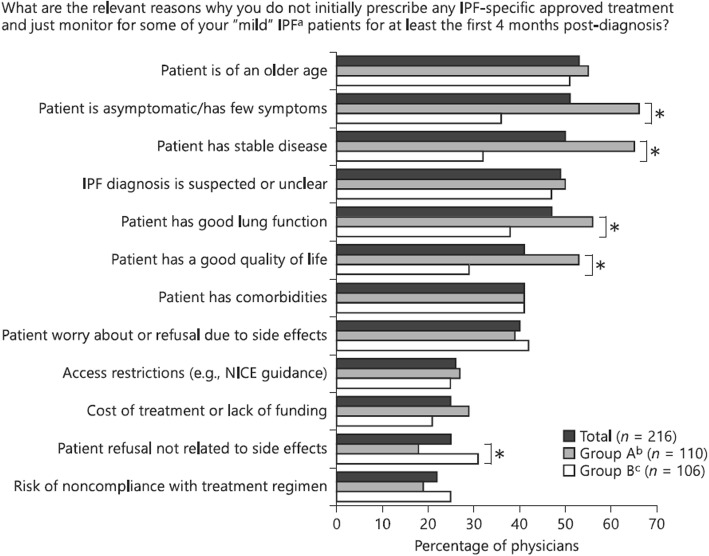
Reasons given by pulmonologists for not treating with “mild” IPF in an international survey [48]. *p < 0.05. a. IPF was defined as “mild” by the physician. b. Physicians who monitored the majority of patients with IPF for > 4 months after diagnosis before initiating treatment. c. Physicians who initiated antifibrotic treatment within 4 months of diagnosis in the majority of patients with IPF. NICE, National Institute for Health and Care Excellence (in the UK). Data from survey of pulmonologist in Canada, France, Germany, Italy, Spain and the UK. Republished with permission of Respiration, from Identifying barriers to idiopathic pulmonary fibrosis treatment: a survey of patient and physician views, Maher et al., doi: 10.1159/000490667, 2018; permission conveyed through Copyright Clearance Center, Inc.

Although the provision of antifibrotic therapies is increasing [36], their use in patients with IPF is far from universal. Data from Europe and the US suggest that only approximately 60% of patients with IPF are receiving nintedanib or pirfenidone [47–52]. Reasons for patients with IPF not receiving antifibrotic therapy include perceptions on the part of the physician that the patient’s disease is “mild” or “stable” and so does not warrant therapy, a lack of confidence in the diagnosis of IPF, access/reimbursement issues, and concerns over the adverse effects of antifibrotic drugs [47, 48] (Fig. 7). A recent international survey of pulmonologists and patients found almost a quarter of the pulmonologists were more concerned about the side-effects of drug therapy than the risk of disease progression, while the patients reported that they wanted more information about the prognosis of their disease and pharmacological treatment options, and were more concerned about preventing disease progression than avoiding medication side-effects [48]. Interestingly only 57% of the patients surveyed recalled being told at their initial visit that IPF is progressive, and fewer than half recalled being informed about treatment options. Pulmonologists who waited > 4 months between diagnosis and initiation of treatment in the majority of patients with IPF were less comfortable discussing the prognosis of the disease with their patients and had less belief in the efficacy of antifibrotic drugs than pulmonologists who initiated antifibrotic therapy in the majority of patients within 4 months of diagnosis. This suggests that the care of patients with IPF might be advanced by training pulmonologists to better understand the balance between the risks and benefits of antifibrotic therapy and on how to communicate to their patients the progressive and invariably fatal nature of IPF and the potential value of taking an antifibrotic therapy.

Prompt treatment of IPF is critical to preserving patients’ lung function, reducing the risk of acute exacerbations and improving outcomes. Arguably, antifibrotic therapy should be initiated in all patients with IPF given that the course of disease for an individual cannot be predicted at diagnosis and the overall prognosis of untreated IPF is dismal. Physicians have a key role to play in explaining to patients that the aim of drug therapy is to slow the progression of their disease and that decline in FVC while taking a drug may not indicate a failure of treatment. While a small proportion of patients may make an informed decision not to proceed with treatment, physicians need to ensure that patients are making that decision based on an understanding that IPF is a progressive, irreversible and fatal disease for which early intervention can improve outcomes.

### Managing side-effects of antifibrotic therapies

Antifibrotic therapies are associated with gastrointestinal adverse events [26, 53–55]. The most common side-effect of nintedanib is diarrhea, which was reported in 62.4% of subjects treated with nintedanib compared with 18.4% of subjects given placebo, in the INPULSIS trials, but led to permanent treatment discontinuation in only 4.4% of the nintedanib group [53]. Pirfenidone is more commonly associated with nausea and decreased appetite than with diarrhea [54, 55] and is recommended to be taken during or after a meal to minimize gastrointestinal issues. Pirfenidone is also associated with rash and photosensitivity, so patients should be advised to minimize exposure to the sun and use high-factor sun block. Both nintedanib and pirfenidone have been associated with elevations in liver enzymes. Few data are available on the tolerability of combination therapy with nintedanib and pirfenidone, but it appears to have an adverse event profile consistent with those of the individual drugs [56, 57].

Management of the side-effects that may occur when they take antifibrotic therapy is important to helping patients stay on treatment. Dose adjustment, through treatment interruption and dose reduction, and symptomatic relief of gastrointestinal adverse events using adequate hydration and medications such as loperamide, are recommended to manage side-effects. Importantly, the dose adjustments made to manage side-effects in clinical trials did not reduce the benefits of treatment in decreasing lung function decline [58, 59]. Most patients are able to tolerate antifibrotic therapy, and discontinuations due to adverse events decrease over time [59, 60]. Education is key to patients understanding the role of antifibrotic therapies in reducing disease progression, so that they can make an informed assessment of the benefits of therapy in the context of side effects that may occur.

### Other therapies used in patients with IPF

The latest international treatment guideline gave a conditional recommendation for the use of anti-acid medications in patients with IPF and asymptomatic gastroesophageal reflux disease (GERD) [31]. However, there is no evidence from randomized controlled trials to support this recommendation and the value of anti-acid medications in the treatment for IPF remains the subject of debate [61]. Post-hoc analyses of data from large randomized controlled trials have shown no benefit of anti-acid medications in reducing FVC decline in subjects with IPF, and suggest that the use of such medications may be associated with an increased risk of infections and acute exacerbations [25, 28, 62]. Whether this increased risk is caused by anti-acid medication permitting translocation of bacteria from the upper gastrointestinal tract into the lungs, or is simply a reflection of greater disease severity in patients with IPF who take these medications remains unclear. Sildenafil has been investigated as a treatment for IPF in individuals with severely impaired gas exchange in two clinical trials: versus placebo in STEP-IPF [63] and in combination with nintedanib versus nintedanib alone in the INSTAGE trial [27]. In both these trials, the primary endpoint was not met, but exploratory analyses of secondary endpoints suggested potential benefits of sildenafil; a further trial of sildenafil in combination with pirfenidone in patients with IPF and severely impaired gas exchange (NCT02951429) is ongoing. Other therapies commonly used in the treatment of IPF (e.g. N-Acetylcysteine, steroids, bronchodilators) have not been demonstrated to have efficacy in slowing the progression of IPF. Triple therapy with prednisone, azathioprine and N-Acetylcysteine (but not N-Acetylcysteine alone) was shown in the PANTHER-IPF trial to be harmful to patients with IPF [64, 65].

### A holistic approach to the management of IPF

In addition to anti-fibrotic therapies, patients with IPF benefit from a holistic approach to care that may include pulmonary rehabilitation, symptom management, education and support, vaccinations, management of comorbidities, supplemental oxygen for those with hypoxemia, and palliative care tailored to the needs of the patient and their caregivers [66–68]. Lung transplant is an option for a minority of patients with IPF and patients should be referred for transplant evaluation at an early stage of disease to maximize their chances of meeting eligibility criteria. The possibility of enrolling patients into clinical trials of investigational therapies should also be considered at an early stage.

## Conclusions

Although the course of disease for an individual cannot be predicted at diagnosis, IPF is an inevitably progressive disease with a very poor prognosis. Prompt treatment of IPF is critical to preserving individuals’ lung function, reducing the risk of acute exacerbations and improving outcomes. Pulmonologists may be reluctant to initiate antifibrotic therapy in individuals with IPF whose lung function appears to be stable; nonetheless they have an obligation to explain to patients that their disease is progressive and that therapies are available that slow progression but that cannot reverse fibrosis or improve breathlessness once progression has occurred. Physicians also have a key role to play in helping patients manage side-effects of antifibrotic therapies through education and dose adjustment, thus enabling them to gain the advantages of long-term treatment.

## Data Availability

Not applicable.

## Abbreviations

CASA-Q: Cough and Sputum Assessment Questionnaire
COPD: Chronic obstructive pulmonary disease
FVC: Forced vital capacity
GERD: Gastroesophageal reflux disease
HRCT: High-resolution computed tomography
ILD: Interstitial lung disease
IPF: Idiopathic pulmonary fibrosis
NICE: National Institute for Health and Care Excellence
SGRQ: St George’s Respiratory Questionnaire
UCSD-SOBQ: University of California San Diego Shortness of Breath Questionnaire
